# Extended cleavage specificity of human neutrophil cathepsin G: A low activity protease with dual chymase and tryptase-type specificities

**DOI:** 10.1371/journal.pone.0195077

**Published:** 2018-04-13

**Authors:** Michael Thorpe, Zhirong Fu, Gurdeep Chahal, Srinivas Akula, Jukka Kervinen, Lawrence de Garavilla, Lars Hellman

**Affiliations:** 1 Department of Cell and Molecular Biology, Uppsala University, Uppsala, The Biomedical Center, Uppsala, Sweden; 2 Fraunhofer USA Center for Molecular Biotechnology, Newark, United States of America; 3 GDL Pharmaceutical Consulting and Contracting, Downingtown, Pennsylvania, United States of America; Universität Stuttgart, GERMANY

## Abstract

Human neutrophils express at least four active serine proteases, cathepsin G, N-elastase, proteinase 3 and neutrophil serine protease 4 (NSP4). They have all been extensively studied due to their importance in neutrophil biology and immunity. However, their extended cleavage specificities have never been determined in detail. Here we present a detailed cleavage specificity analysis of human cathepsin G (hCG). The specificity was determined by phage display analysis and the importance of individual amino acids in and around the cleavage site was then validated using novel recombinant substrates. To provide a broader context to this serine protease, a comparison was made to the related mast cell protease, human chymase (HC). hCG showed similar characteristics to HC including both the primary and extended specificities. As expected, Phe, Tyr, Trp and Leu were preferred in the P1 position. In addition, both proteases showed a preference for negatively charged amino acids in the P2´ position of substrates and a preference for aliphatic amino acids both upstream and downstream of the cleavage site. However, overall the catalytic activity of hCG was ~10-fold lower than HC. hCG has previously been reported to have a dual specificity consisting of chymase and tryptase-type activities. In our analysis, tryptase activity against substrates with Lys in P1 cleavage position was indeed only 2-fold less efficient as compared to optimal chymase substrates supporting strong dual-type specificity. We hope the information presented here on extended cleavage specificities of hCG and HC will assist in the search for novel in vivo substrates for these proteases as well as aid in the efforts to better understand the role of hCG in immunity and bacterial defence.

## Introduction

Neutrophils constitute 50 to 75% of all leukocytes in human blood and are thereby the most abundant human white blood cell [1, 2]. They are short-lived cells, which use both oxygen and non-oxygen dependent mechanisms as well as neutrophil extracellular traps (NETs), which are web-like structures consisting of nuclear DNA, histones and granule components, to kill bacteria [3–5]. In addition to secretory vesicles, three types of cytoplasmic granules are found within neutrophils, the specific granules, the azurophilic granules, and the gelatinase granules [6, 7]. These granules contain a number of different components and the majority of them are involved in bacterial defence. Several abundant antibacterial peptides including cathelicidins and defensins, are found in these granules together with other antibacterial substances such as bacterial permeability increasing protein (BPI), lysozyme, lactoferrin and several serine proteases [6–8]. Four such active proteases have been identified: cathepsin G (CG), N-elastase (NE), proteinase 3 (PR3) and neutrophil serine protease 4 (NSP4) [9–12]. Human neutrophils also express a close homologue to these proteases, azurocidin, which is a potent antibacterial protein that lacks proteolytic activity [13]. This loss of proteolytic activity is caused by mutations in two of the three amino acids in the catalytic triad of the active site [13]. These four proteases and protease homologs (azurocidin) are primarily considered neutrophil specific proteases, however CG is also expressed in other cells, particularly macrophages, upon infection [14]. Human CG but not its mouse and rat counterparts, is also expressed in mast cells and low levels of these proteases are also found in a subpopulation of monocytes due to their expression during early stages of myelo-monocyte development.

The four active neutrophil proteases have a number of different functions and also an array of potential substrates. Knock out experiments show that several of these enzymes are important for bacterial and fungal defence [15–21]. Two potential bacterial targets have been identified as flagellin of *Pseudomonas aeruginosa* and the outer membrane protein A of *E*. *coli* [22, 23]. Furthermore, knocking out both CG and NE impairs the elimination of *Mycobacterium bovis* lung infections in mice [20]. In the same study, therapeutic administration of liposomes loaded with NE and CG also resulted in significant enhancement of mycobacterial protection [20]. Experimental infections using another mycobacterial species, the human pathogen *Mycobacterium tuberculosis*, also show reduced survival rates of both single (CG) and double knock out mice (NE+CG) without having any direct effect on bacterial replication themselves. However, there are increased levels of several inflammatory cytokines including TNF-α, IL-6 and MIP2, in the knock out mice during early infection indicating dys-regulated cytokine expression [21]. Modulation of the expression of these same cytokines was also observed with another pathogen *Pseudomonas aeruginosa*. However, a strong down-regulation in the knock out animals was seen, highlighting a complex role of these proteases in cytokine regulation [24]. Interestingly, CG and NE are directly involved in Fc receptor dependent activation of neutrophils by immune complexes [25]. The activation results in an integrin dependent clustering of neutrophils, and in the absence of NE and CG the knock out mice show a severe defect in MIP-2 secretion and oxygen radical production [25]. In line with the role of these enzymes in cell adhesion and clustering, CG is also involved in arterial myeloid cell recruitment by strengthening adhesion of the cells during high shear flow [26].

The killing ability of neutrophils within different gram positive bacteria as well as between gram positive and negative bacteria differs markedly. For example *Staphylococcus aureus* is killed primarily by oxygen dependent mechanisms involving reactive oxygen species (ROS) whereas the killing of another gram positive bacteria *Streptococcus pneumonia* is independent of ROS but dependent on phagocytosis and complement mediated opsonisation [18]. This particular study also showed that killing of the *Streptococcus* was dependent on active serine proteases. Here the effect seemed to be a combined protease response as individual inhibition of specific proteases did not lead to a loss in killing activity (NSP-4 was not analysed) but inhibiting all three reduced killing to baseline [18]. Both NE and CG appear to have non-redundant roles in lung protective immunity against *Streptococcus pneumonia* [19]. Patients with Papillon-Lefèvre syndrome show a particular severe form of periodontitis due to bacterial infection with *Actinobacillus actinomycetemcomitans* and *Porphyromonas gingivalis*. These patients lack cathepsin C, which is an enzyme involved in the activation of all the neutrophil proteases. The role of the neutrophil protease here is related to neutralizing bacterial toxins and also activating one of the neutrophil antibacterial peptides, hCAP-18 [27]. N-elastase and hCG may also have indirect antimicrobial effects by their recently identified effect on blood coagulation. These two neutrophil proteases can enhance coagulation by the cleavage of an inhibitor of the tissue factor pathway (TFPI), an effect that is strongly potentiated by histones and DNA released by the NETs [28]. The bacteria are subsequently trapped by the coagulation in small blood vessels and are thereby inhibited from entering tissues, which results in a decrease in bacterial numbers [28]. Human cathepsin G can also activate coagulation factor VIII, which may contribute to the enhanced coagulation in areas of infection where neutrophils accumulate [29]. Finally, in support of the role of these proteases in coagulation, both the single (CG) and double knock out mice (NE+CG) show a bleeding time that is twice as long as the wild-type mice [21]. The role of mammalian neutrophils in coagulation links this aspect to innate immunity, which is similar to what is observed in many invertebrate species where coagulation has a central role in antimicrobial immunity [30].

Of the four active neutrophil serine proteases hCG, is one of the more abundant enzymes, and is probably the most extensively studied protease of these four [31]. A relatively detailed analysis of hCG has previously been performed using peptide libraries where hCG was compared with its mouse counterpart mCG [32]. Human cathepsin G but not mCG displayed a dual specificity as both a chymase and a tryptase, where the later activity favoured lysine over arginine [31–33]. The lower activity for human but not mouse CG, compared to human chymase (HC), has been attributed towards the widening of its active site, which may also explain the broader specificity seen with hCG [32]. Although hCG and the other neutrophil proteases are relatively well characterized there are a number of important unanswered questions concerning them. Their extended specificities have never been determined in detail and almost no quantitative information concerning the importance of various positions in and around the cleavage site has been presented. Such information can be used to increase the resolution during screenings of the human genome and genomes of pathogens sensitive to these enzymes in order to identify novel in vivo substrates. This would serve as a tool for understanding the general roles of them in immunity with a particular focus in bacterial defence.

In order to close this gap in our understanding of these enzymes, here we present a detailed analysis of the extended cleavage specificity of hCG. Substrate phage display was used in order to obtain an estimate of the most favoured (up to 9) amino acids in a region surrounding the cleavage site. These sequences were then verified and/or modified in order to pinpoint specific amino acid involvement in this cleavage. Our results showed that hCG is approximately 10 times less active than the HC at a similar molar ratio. As expected, hCG preferred Tyr and Phe in its P1 position but also cleaved efficiently after Trp and Leu. Interestingly hCG also showed a preference for negatively charged amino acids in the P2´position, which is similar to HC as well as the functional homologue of HC in mice, mouse mast cell protease 4 (mMCP-4) [34]. We also verified the activity of hCG on Lys containing substrates in the P1 position. Our results showed that hCG displayed only 2 times lower activity on Lys compared to Tyr and Phe, which clearly shows that it contains tryptase-type cleavage specificity after positively charged Lys. However, it showed little activity after Arg. Finally, we also showed that HC has a low tryptase-type activity on Arg, which was approximately 20 times lower than its favourable chymase activity.

Recently an alternative method to study cleavage specificity has been developed using a peptide library and subsequent analysis of cleavage products by mass spectroscopy (MS) [35, 36]. In order to study the advantages and disadvantages of this method we have analyzed the cleavage of the identified consensus cleavage sites using our novel thioredoxin-based cleavage assay, with four different human neutrophil proteases: hCG, N-elastase, proteinase 3 and NSP4. Here, we saw that for proteases with a relatively broad specificity, such as N-elastase and proteinase 3, the peptide library/MS method provided similar results to our analyses. However, for more specific proteases such as hCG, the phage display results were much more reliable as they originate from peptide libraries several orders of magnitude larger in complexity compared to the proteomics method used.

## Results

### Human cathepsin G and human mast cell chymase

**Fig 1:**
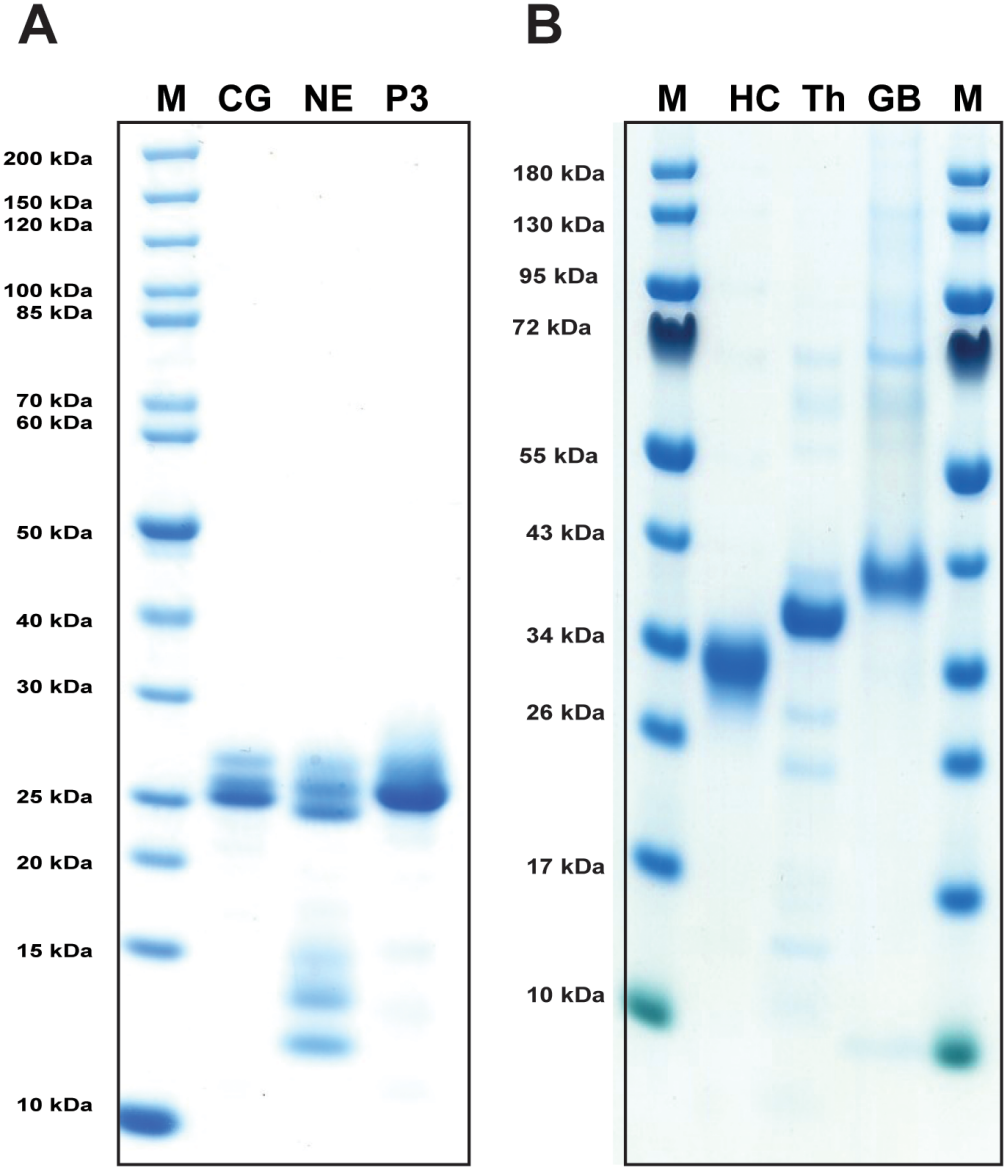
Analysis of the human cathepsin G preparation used in the determination of the extended cleavage specificity. The enzyme had previously been purified from blood neutrophils and the purity of the preparation was determined by separation on SDS-PAGE and visualized with Coomassie Brilliant Blue staining. In panel A commercial preparations of two additional neutrophil proteases N-elastase (NE) and proteinase 3 (P3) were also included in the figure for comparison. Proteinase 3 and hCG were intact after short term storage at +4°C. However, N-elastase showed several degradation products indicating self-cleaving activity. In panel B three additional enzymes, the human mast cell chymase (HC), human thrombin (Th) and human granzyme B (GB) were included as they were used as reference proteases in the chromogenic and recombinant substrate assays.

Human cathepsin G used for both the phage display analysis and subsequent verification by the recombinant substrates was a commercially available preparation from BioCentrum (Krakow, Poland) that had been purified by several steps from human peripheral blood neutrophils (Fig 1A). Recombinant HC was also used for a comparative analysis of activity and extended specificity (Fig 1B). This enzyme was produced via baculovirus infected insect cells as previously described [37]. Human N-elastase and proteinase 3 were also included for comparison (Fig 1A) and both were also commercially available from peripheral blood neutrophils. In Fig 1A both hCG and proteinase 3 are relatively stable after purification whereas N-elastase showed unavoidable self cleavage at a low rate even when stored at +4°C. The preparations of human thrombin (Th) and human granzyme B (GB) were also included (Fig 1B) as they were used as reference proteases in the chromogenic and recombinant substrate assays.

### Chromogenic substrate assay

**Fig 2:**
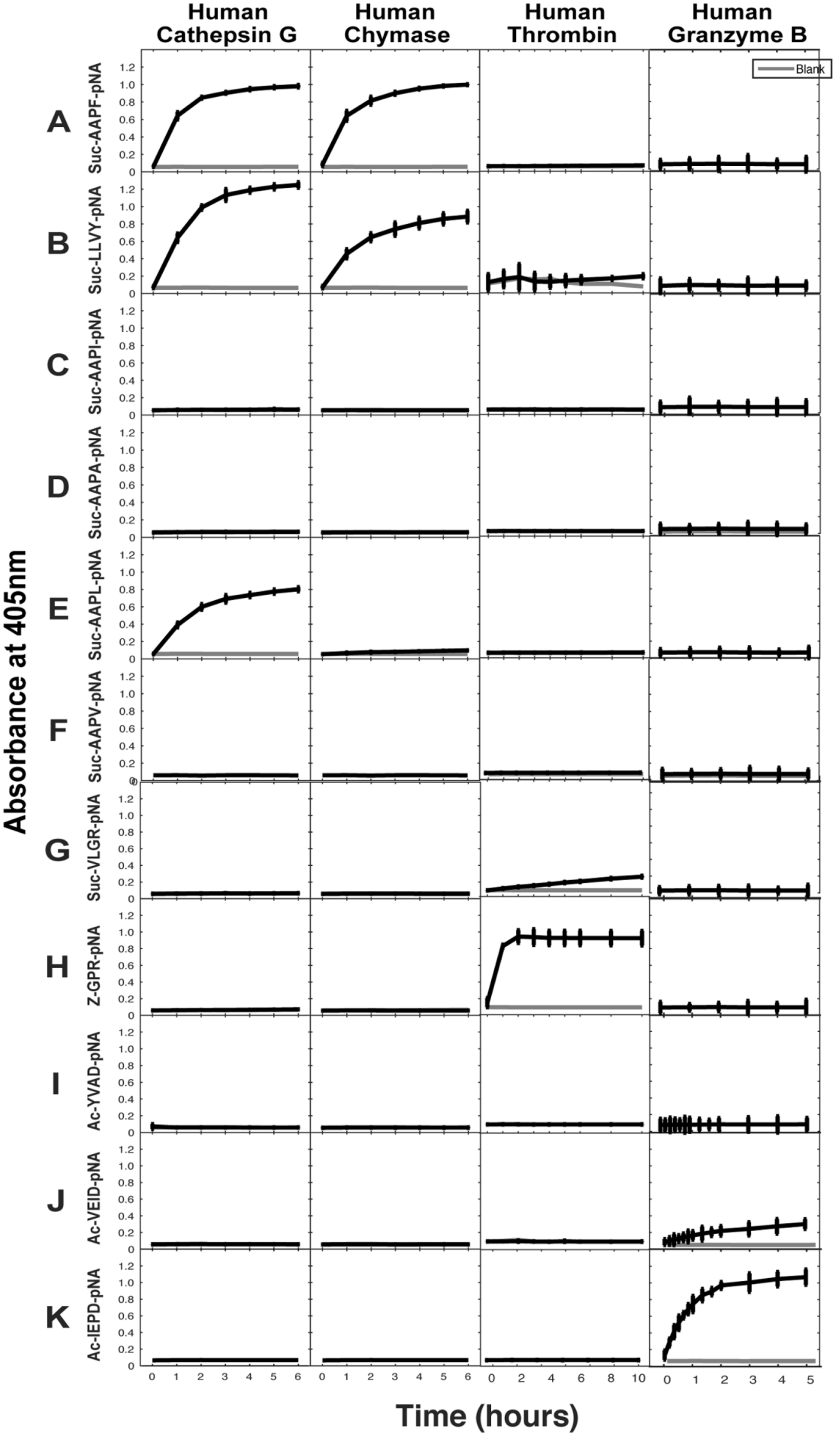
Chromogenic substrate assay. A panel of different chromogenic substrates was used to determine the primary specificity of hCG and HC. The panel included different chymase, elastase, tryptase and aspase substrates. The amino acid sequences of the substrates are listed at the left side of the figure. Human thrombin and human granzyme B were included as reference enzymes for tryptase and asp-ase activities, respectively.

A large panel of different chromogenic substrates was used to determine the primary specificities of hCG and HC. In order for complete specificity coverage the panel included different chymase, elastase, tryptase and asp-ase substrates. In Fig 2 hCG cleaved the classical chymase substrates with Phe and Tyr efficiently as well as the substrate with a Leu in the P1 position. However, all other substrates remained uncleaved including the two tryptase substrates (Z-GPR-pNA, Suc-VLGR-pNA) with an Arg in the P1 position (Fig 2 first column). The HC also cleaved the Phe and Tyr substrates efficiently but not the P1 Leu containing substrate highlighting an apparent difference between the two enzymes under these conditions (Fig 2 second column). As further controls for this assay, two additional serine proteases, thrombin and granzyme B, with tryptase and asp-ase specificities, respectively were tested (Fig 2 panels 3 and 4). Importantly, approximately 10 times more enzyme was needed for hCG indicating a much lower activity of this enzyme compared to the HC (100 ng HC compared to 1000 ng for hCG).

### Determination of the extended cleavage specificity by substrate phage display

To obtain a more complete view of the extended specificity of hCG we performed unbiased screening for the most favoured targets by substrate phage display. The phage library used to determine the extended cleavage specificity of hCG contains approximately 5x10^7^ phage clones. Each phage clone expresses a unique sequence of 9 random amino acids (nonamer) on their surface, followed by a His_6_-tag in the C-terminus of capsid protein 10. The phages are subsequently immobilized on Ni-NTA agarose beads via interactions with the His_6_-tag. The purified hCG was used to screen the phage library for peptides susceptible to cleavage. After the first selection step (biopanning), the released phages, which are cleaved in their unique random region, were amplified in *E*. *coli* and subjected to additional biopannings. Selections of phages susceptible to cleavage by the protease, were performed over 5 biopannings, after which hCG induced the release of 71 times more phages compared to a PBS control (data not shown).

**Fig 3:**
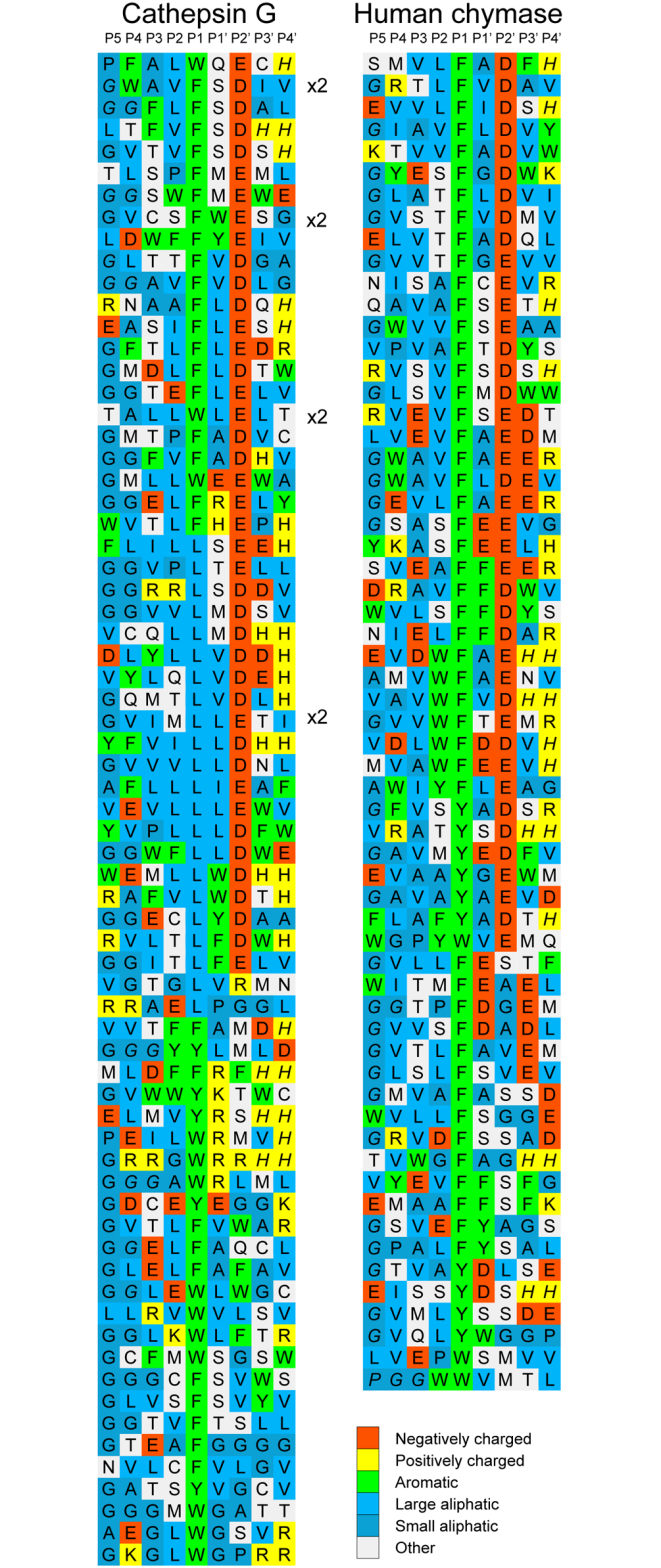
Phage displayed nonamers susceptible to cleavage by hCG after five biopannings. After the last selection step, phages released by proteolytic cleavage were isolated and the sequences encoding the nonamers were determined. The general sequence of the T7 phage capsid proteins are PGG(X)_9_HHHHHH, where (X)_9_ indicates the randomized nonamers. The protein sequences were aligned into a P5-P4´ consensus, where cleavage occurs between positions P1 and P1´. The amino acids are colour coded according to the side chain properties as shown in a separate panel in the bottom of the figure. The sequences with one aromatic amino acid (potential cleavage site) are aligned first, followed by sequences containing two, three or four aromatic amino acids. It is not a perfect alignment but so far the best that has been obtained by any method.

**Fig 4:**
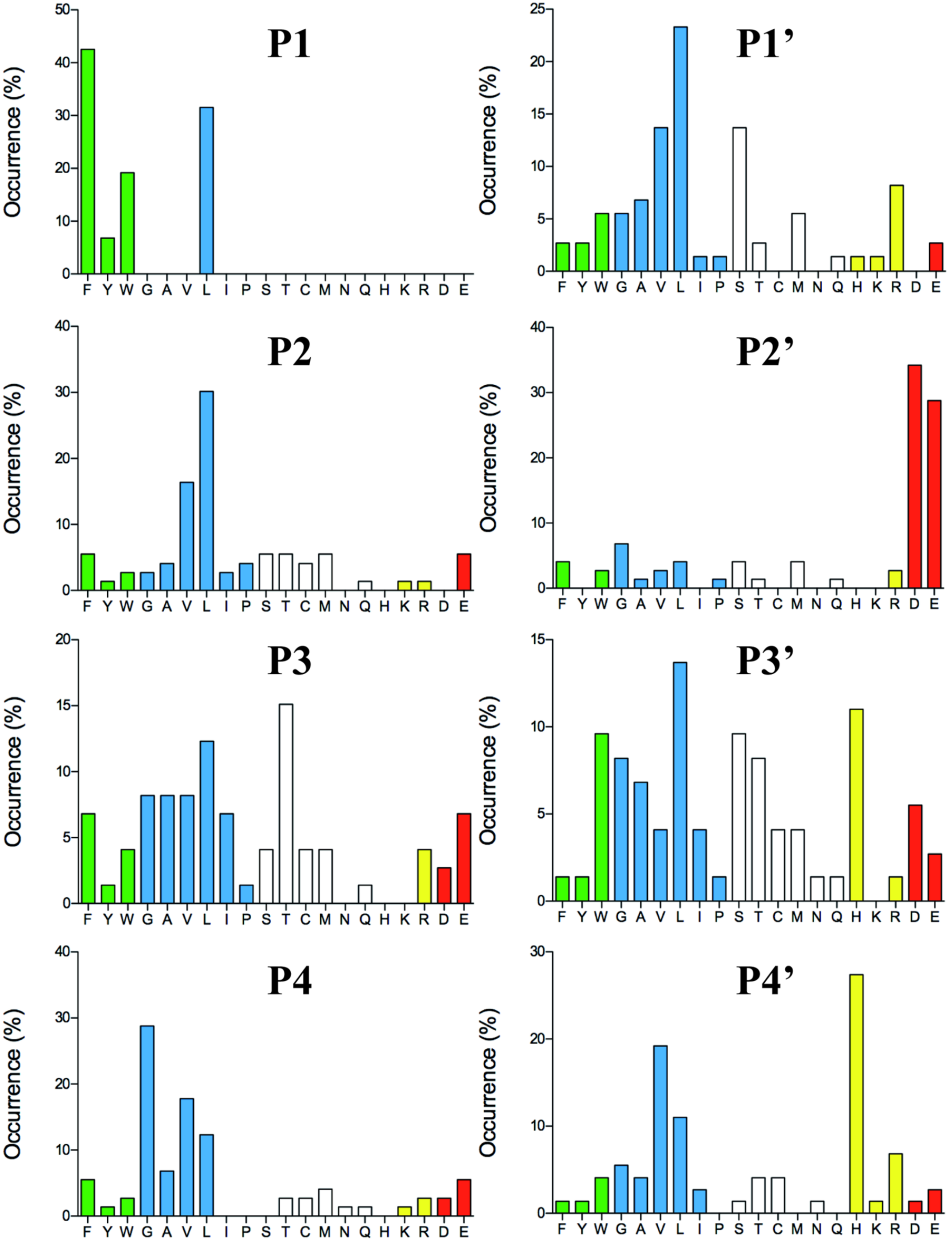
Distribution of amino acids in positions P4 to P4´ in phage displayed nonamers cleaved by hCG after five biopannings. Based on the alignment (Fig 3) the percentage of each amino acid present in each position P4 to P4´ as calculated. The amino acids are ordered from left to right: aromatic, aliphatic, hydrophilic, basic (positively charged) and acidic (negatively charged).

After the last biopanning, 120 individual phage clones were isolated and the sequences encoding the randomly synthesized nona-peptides were determined for 96 of them (one 96 well plate). The nucleotide sequences were then translated into nona-peptides, which were aligned based on the primary cleavage specificity observed from the chromogenic substrate assay (Fig 2). The alignment of the phage display sequences is performed by hand starting with the substrates having only one aromatic amino acid or one Leu. Based on this alignment we then continue using the same basic pattern and align the sequences containing several potential cleavage sites. What we can conclude from this alignment is that in addition to the similar preference of Phe/Tyr/Trp/Leu in the P1 position hCG, like HC [38], has a clear specificity for negatively charged amino acids in the P2’ position. A strong preference for aliphatic amino acids both N and C terminal of the cleavage site was also observed (Figs 3 and 4). Compared to HC a marked difference in the number of Leu in the P1 position was observed (Figs 3 and 4). However we could not observe a high frequency of Arg or Lys containing phages that could indicate tryptase activity. The phage display method primarily selects the most favourable substrates and secondary specificities can thereby easily remain undetected, a fact that needs to be taken into consideration when analysing complex enzymes with several specificities. The phage display technology does not allow for the identification of the exact cleavage site. However, by combining the information from chromogenic substrates and the verification by the recombinant substrates described in the next section we obtain a very accurate picture of the cleavage preference.

### Verifying the consensus sequence by the use of a new type of recombinant protein substrate

**Fig 5:**
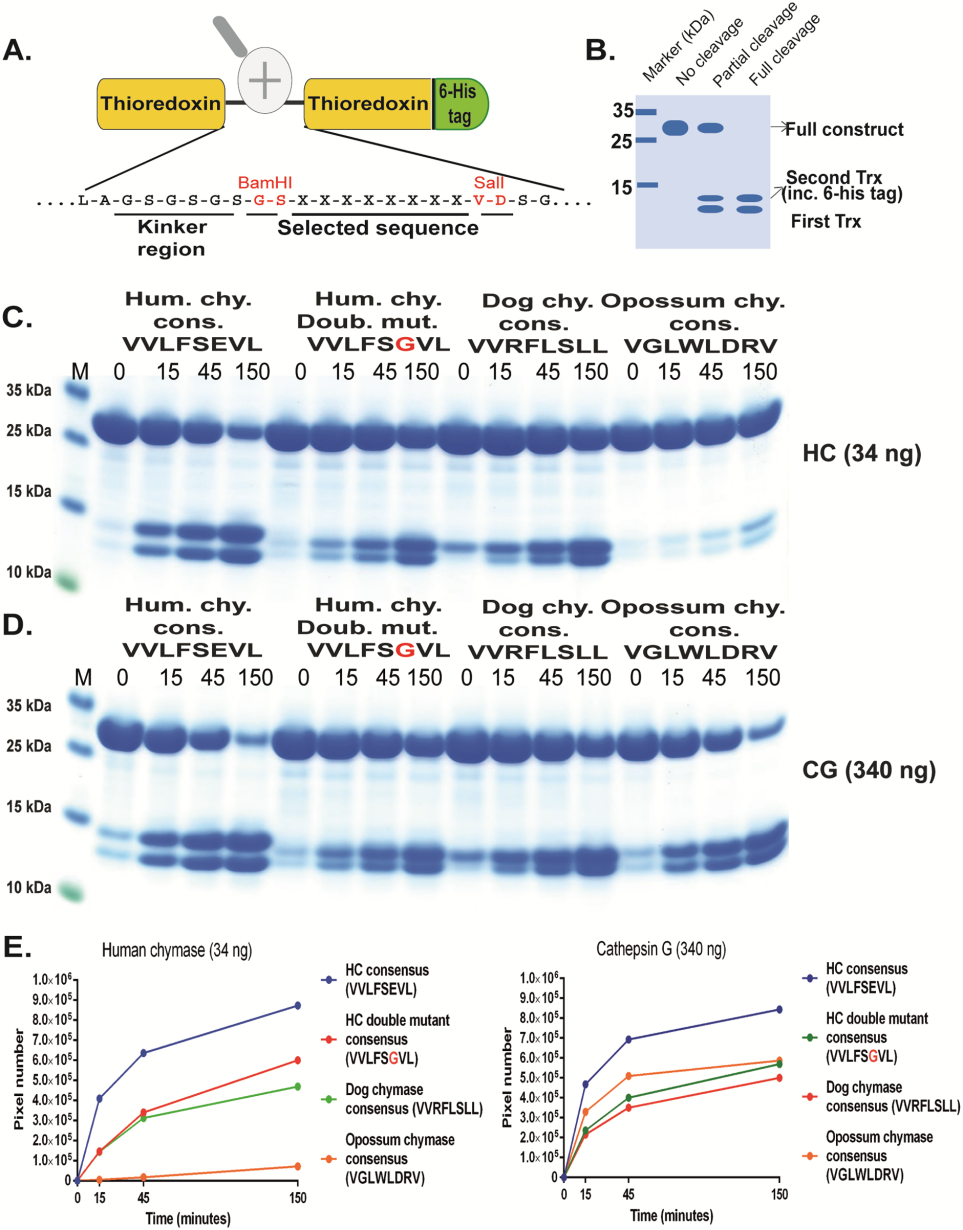
Analysis of the cleavage specificity by the use of recombinant protein substrates. Panel A shows the overall structure of the recombinant protein substrates used for analysis of the efficiency in cleavage by the hCG and HC. In these substrates two thioredoxin molecules are positioned in tandem and the proteins have a His_6_-tag positioned in their C termini. The different cleavable sequences are inserted in the linker region between the two thioredoxin molecules by the use of two unique restriction sites, one Bam HI and one SalI site, which are indicated in the bottom of panel A. In panel B a hypothetical cleavage is shown to highlight possible cleavage patterns. Panels C and D shows the cleavage of 4 different substrates by hCG and HC, the HC consensus obtained from phage display, the consensus of a HC double mutant, the dog chymase consensus and the opossum chymase consensus [34, 46, 52]. The name and sequence of the different substrates are indicated above the pictures of the gels. The time of cleavage in min is also indicated above the corresponding lanes of the different gels. The uncleaved substrates have a molecular weight of approximately 25 kDa and the cleaved substrates appear as two closely located bands with a size of 12–13 kDa. In panel E we show the results from scanning of the gels where on e clearly can see the effect of P2´negative charge for both HC and hCG. The gel pictures in panels C and D were densitometrically scanned and the result was presented as two separate diagrams in panel E, one for HC and one for hCG.

**Fig 6:**
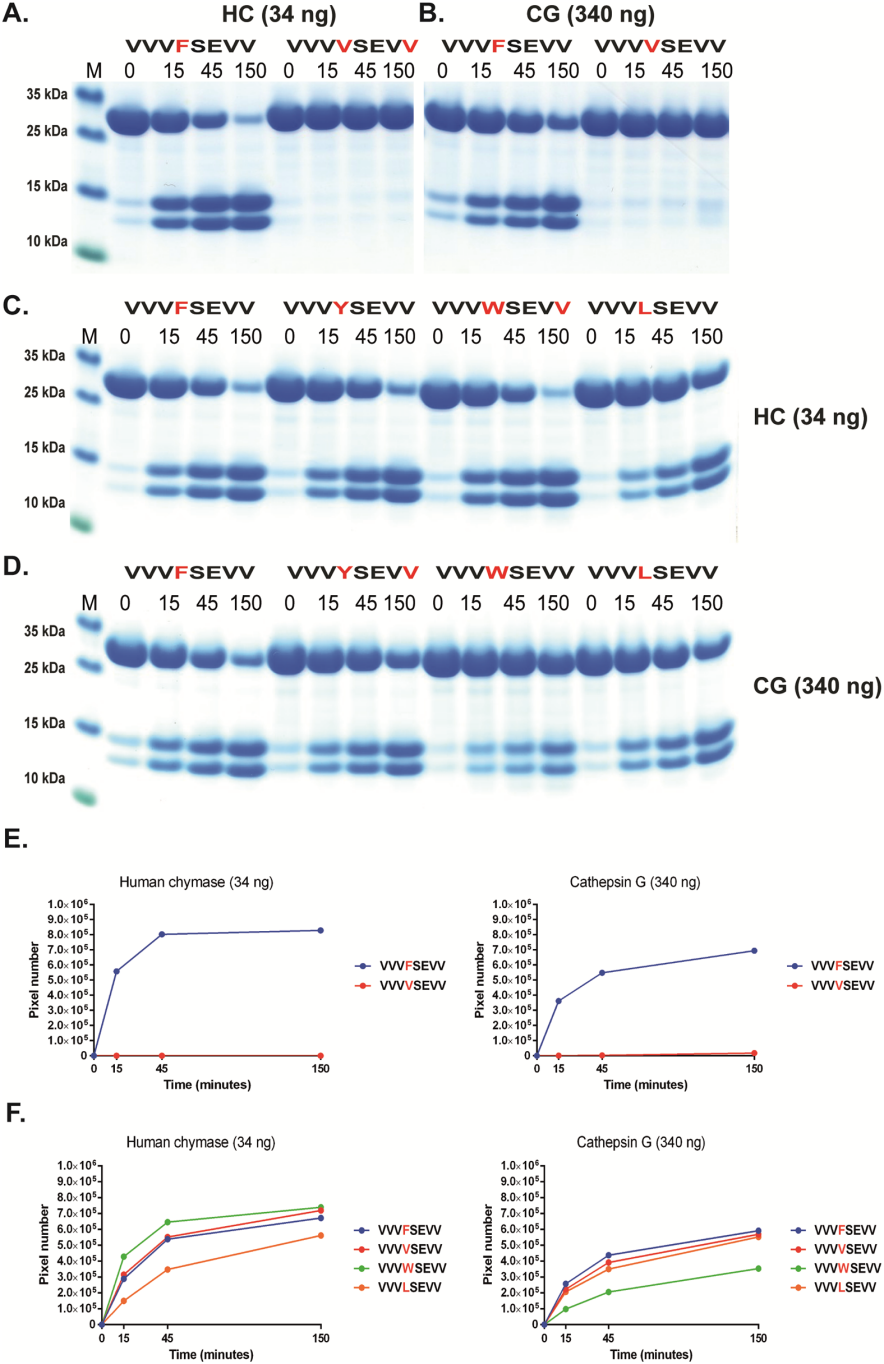
Analysis of the cleavage specificity by the use of recombinant protein substrates. Panels A and B show the cleavage of two substrates, one containing Phe in the P1 position and one where this residue has been exchanged for a Val residue, thereby the construct does not contain any aromatic amino acids or a Leu serving as a negative control. Panels C and D show the analysis of the chymotrypsinogen-like P1 preference for the two enzymes with substrates having different aromatic amino acids or Leu in the P1 position. Panels E and F show an analysis of the P1´and P2´specificities. The sequences of the different substrates are indicated above the pictures of the gels. The time of cleavage in min is also indicated above the corresponding lanes of the different gels. The gel pictures in panels A, B, C and D were densitometrically scanned and the result was presented as two separate diagrams in panels E and F.

**Fig 7:**
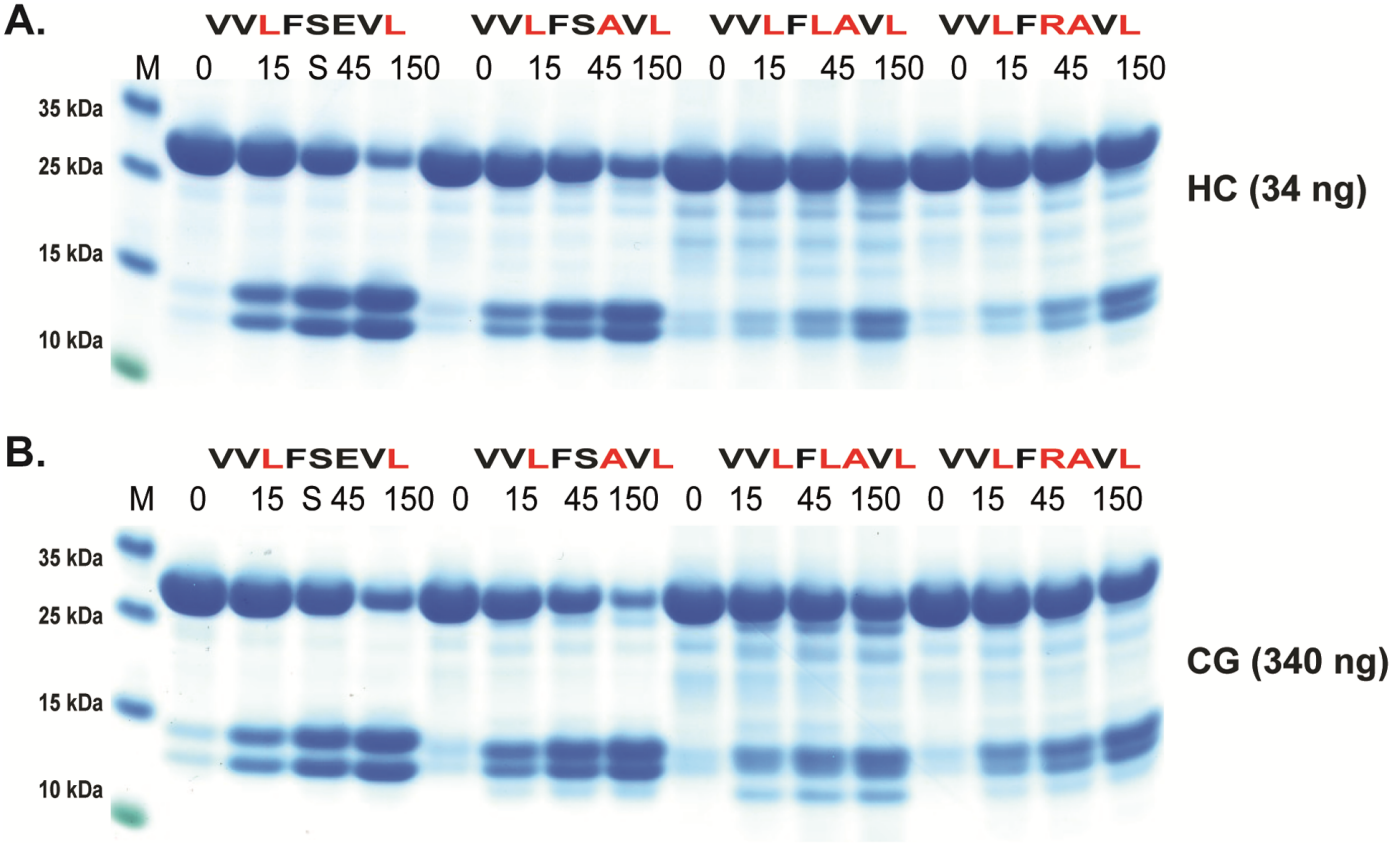
Analysis of the cleavage specificity by the use of recombinant protein substrates. Panels A and B show an analysis of the P1´and P2´specificities. The sequences of the different substrates are indicated above the pictures of the gels. The time of cleavage in min is also indicated above the corresponding lanes of the different gels.

**Fig 8:**
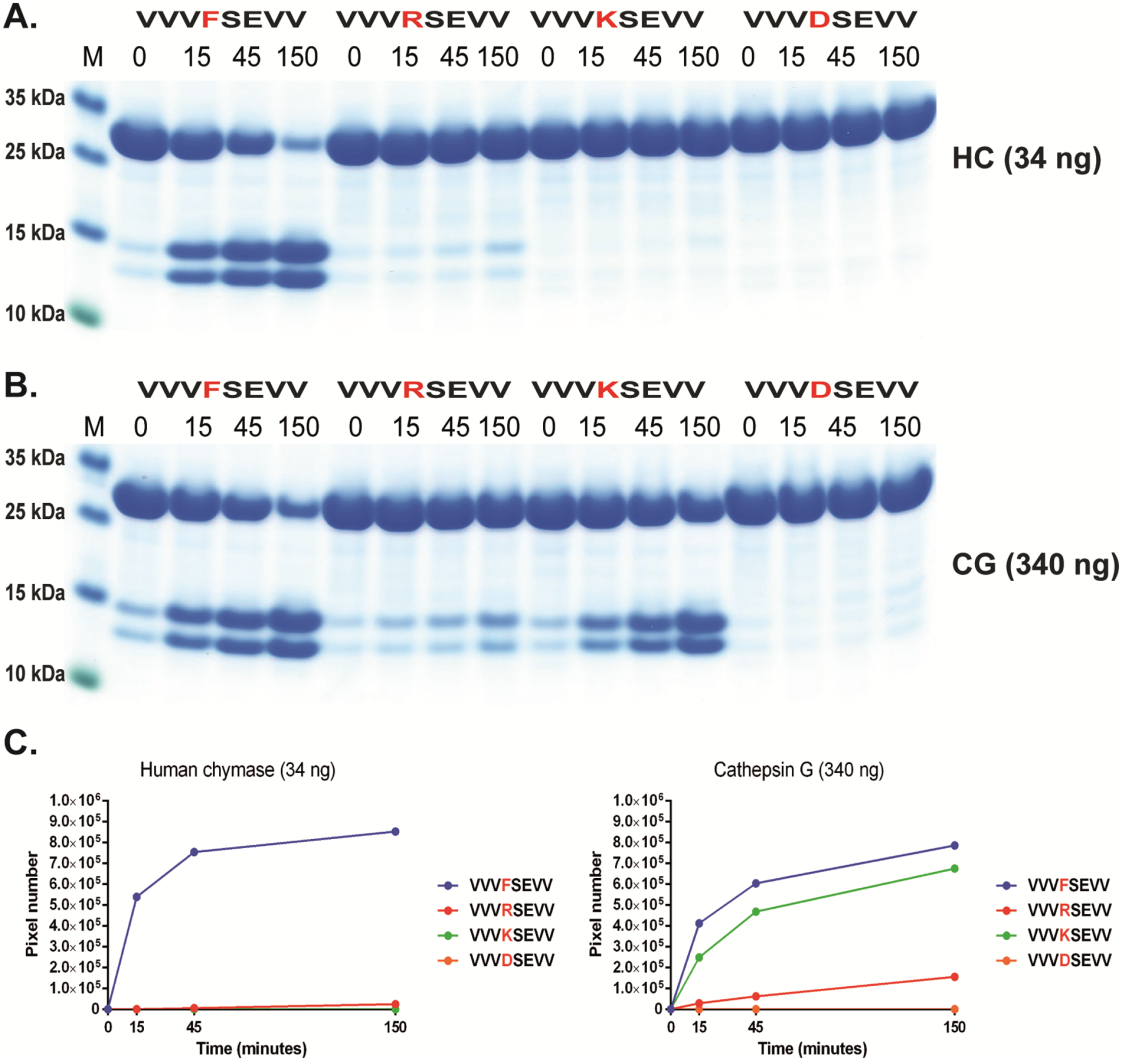
Analysis of the potential tryptase activity by HC and hCG using recombinant protein substrates. Using the Phe substrate (Fig 6) as a reference to determine the cleavage rate by HC and hCG on three substrates having Arg, Lys or Asp in the P1 position. The sequences of the different substrates are indicated above the pictures of the gels. The time of cleavage in min is also indicated above the corresponding lanes of the different gels. The gel pictures in panels A and B were densitometrically scanned and the result was presented as two separate diagrams in panel C, one for HC and one for hCG.

**Fig 9:**
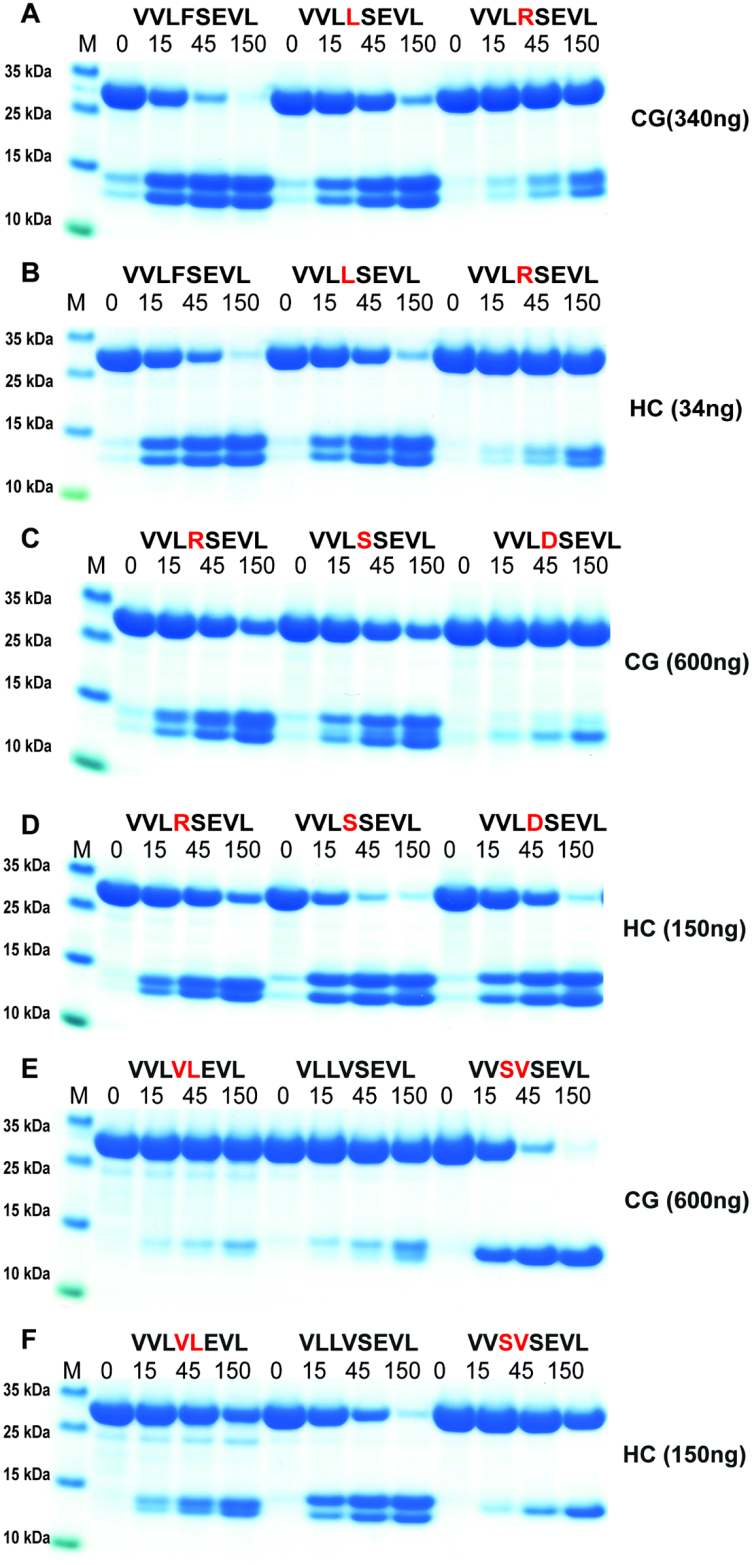
Analysis of the cleavage specificity by the use of recombinant protein substrates. An analysis of the effect of changing amino acids in and around the cleavage site on the cleavage by HC and hCG. The sequences of the different substrates are indicated above the pictures of the gels. The time of cleavage in min is also indicated above the corresponding lanes of the different gels.

**Fig 10:**
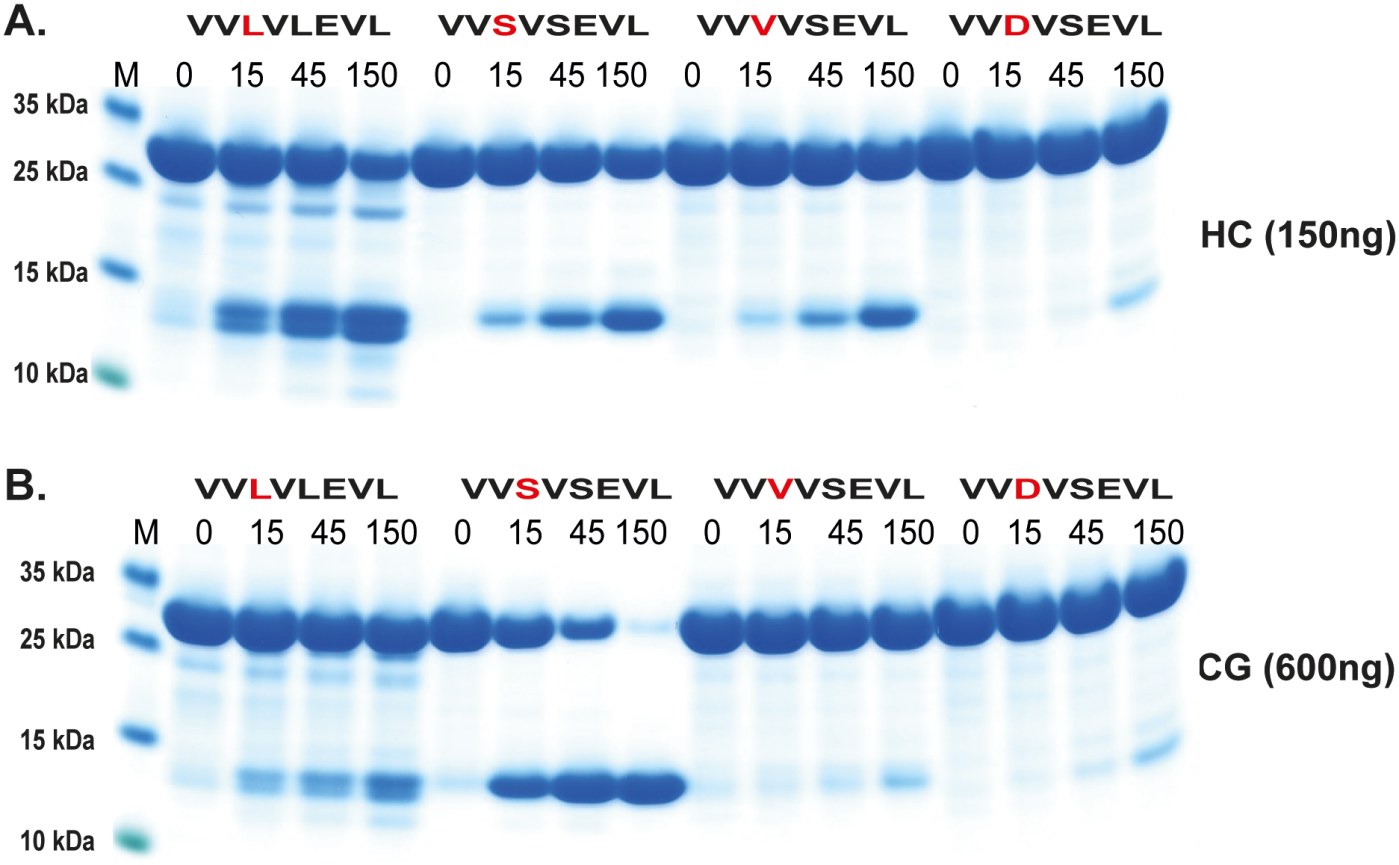
Analysis of the effect of particular amino acids in the P6 position for the cleavage by hCG. The sequences of the different substrates are indicated above the pictures of the gels. The time of cleavage in min is also indicated above the corresponding lanes of the different gels.

In order to verify the results from the phage display analysis, a new type of recombinant substrates that has been validated in a number of previous studies was used [39–45]. The consensus sequence obtained from the phage display analysis was inserted into a linker region between two *E*.*coli* thioredoxin (Trx) molecules by ligating a double stranded oligonucleotide encoding the cleavable sequence into a BamHI and a SalI site of the vector construct (Fig 5A). For purification purposes a His_6_-tag was added to the C-terminal of the second Trx protein (Fig 5A). A number of related and unrelated substrate sequences were also produced with this system, by ligating the corresponding oligonuclotides into the BamHI/SalI sites of the vector. All of these substrates were expressed as soluble proteins in *E*.*coli* and purified on IMAC columns to obtain a protein with a purity of 90–95%. These recombinant proteins were subsequently used to study the preference of hCG and HC for these different sequences (Figs 5–10). Previously we have used several such 2xTrx clones encoding the consensus sequences obtained from phage display analyses of the HC, a HC double mutant (Arg143Gln + Lys192Met) as well as for the dog and opossum mast cell chymases [38–40, 46]. This set of substrates was used for an initial reference and comparison between HC and hCG. In Fig 5 the preference for negatively charged amino acids in the P2´position for both proteases as seen from the phage display analysis was confirmed. We could also see that hCG was more tolerant to Trp in the P1 position in comparison to the HC. However, these four substrates differ in a few additional positions surrounding the cleavage site, which may influence the rate of cleavage.

Based on both chromogenic substrate analyses for hCG (Fig 2) and the phage display (Fig 3), it was apparent that hCG also efficiently cleaves after Leu in the P1 position. As all four of the consensus substrates analysed in the 2xTrx system (Fig 5) contained both aromatic amino acids and several Leu residues it was difficult to obtain an accurate estimate of the preference for the four potential preferred P1 residues, Phe, Tyr, Trp and Leu. We therefore decided to make a new set of 2xTrx substrates with only single aromatic amino acids containing, Phe, Tyr or Trp in the cleavable sequence (Figs 6, 7 and 8). Here it was important to ensure that none of the surrounding amino acids in the 2xTrx substrate could serve as a cleavable P1 site. A substrate lacking Phe, Tyr, Trp and Leu was therefore constructed and analysed using both HC and hCG (Fig 6A and 6B). This substrate has Val in the positions where aromatic amino acids or Leu was originally located. In Fig 6A and 6B both proteases did not cleave this control P1 Val substrate whereas the substrate with Phe in the same position was efficiently cut, which once again confirmed the P1 specificity of the enzyme. In panel D, hCG preferred substrates with Phe over Tyr or Leu in the P1 position by a factor of less than 2 and over Trp with a factor of 2–3 (Fig 6D). In contrast the HC cleaved all three substrates with aromatic amino acids almost to the same extent (Fig 6C). The cleavage rate for the Leu substrate was only slightly lower (2–3 times) than for the three aromatic amino acids (Fig 6C).

Based on above results we concluded that the amino acids surrounding the P1 site were of major importance for the efficiency of cleavage by both enzymes. We therefore decided to study the influence of different amino acids in the P1´and the P2´positions (Fig 7A and 7B). Here the P2´amino acid was changed from Asp to an Ala. Only a very minor effect on the cleavage activity was observed for both enzymes indicating that Ala rather than Gly can replace the negative charge for efficient cleavage. Changing Ser in the P1´position to Leu or Arg resulted in a 3-5-fold drop in cleavage activity for both enzymes, thus also highlighting the importance of the amino acids in this position (Fig 7A and 7B). Interestingly, here the HC showed very similar kinetics for these three substrates although phage display selected few or no sequences with Trp and Leu and no activity on chromogenic substrates containing a Leu in the P1 position (Figs 2 and 3). The lack of cleavage of the chromogenic Leu substrate indicates that the HC is more dependent on downstream residues when cleaving Leu containing substrates compared to substrates with aromatic amino acids in the P1 position.

In addition to having chymase activity, hCG has also been attributed to display tryptase activity with preference for Lys over Arg [31–33]. In order to address this aspect we produced substrates with Arg, Lys and Asp in the P1 position (Fig 8). There were indications of cleavage in the Arg containing substrate although some 10x times less than towards chymase substrates (Fig 8B). However, hCG cleaved the Lys substrate very efficiently almost as equally well as the Tyr substrate. This shows that, in contrast to HC, hCG also is a potent tryptase, but primarily for Lys containing substrates. Interestingly the HC showed some (although very low) activity towards the Arg substrate suggesting that it has some tryptase activity. Importantly, the HC showed no cleavage of the Lys containing substrate revealing a major difference between these two enzymes.

A few additional substrates were tested to obtain a detailed view of the importance of residues surrounding the cleavage site. Using modified versions of the HC consensus in Fig 5 (VVLFSEVL, VVL**L**SEVL, VVL**R**SEVL) we saw in this setting that Leu showed 2-3-fold lower cleavage rate than Phe in the P1 position (Fig 9A). The substrate with P1 Arg following a Leu showed a 3–5 fold reduction in cleavage for both enzymes (Fig 9A and 9B). Exchanging this Arg for a Ser (VVL**R**SEVL to VVL**S**SEVL) resulted in no effect for hCG but an 3–5 fold enhancement for HC (Fig 9 panels C and D). Exchanging the Arg for an Asp (VVL**R**SEVL to VVL**D**SEVL) resulted in a dramatic drop in activity for hCG, so severe that the enzyme cleaved at the Leu in the end of the sequence (Fig 9 panels C and D). The cleavage at the end of the linker region can be deduced from the change in size of the cleavage products (Fig 9 panels C and D). In contrast, the Asp enhanced the cleavage efficiency over Arg for the HC by a factor of 2 (Fig 9 panels C and D). We also made three additional substrates where the Val-Leu-Phe-Ser of the HC consensus has been changed to Val-Leu-Val-Leu, Leu-Leu-Val-Ser or Val-Ser-Val-Ser (VVL**VL**EVL, V**LLV**SEVL, VV**SV**SEVL) (Fig 9E and 9F). Unsurprisingly, very low cleavage was seen with the first two substrates whereas a remarkably high activity for hCG was observed for the third substrate VV**SV**SEVL, resulting in increased cleavage at the Leu residue at the C terminal end of the sequence, indicating that a Ser in the now position P6 is of major importance for efficient cleavage for hCG (Fig 9E). This position does not seem to have the same effect on the HC, which also cleaved at the Leu C-terminal end but at a much lower rate (Fig 9F). Here, the second substrate V**LLV**SEVL was a relatively good target for the HC in contrast to hCG (Fig 9E and 9F). In order to verify the importance of a Ser in the P6 position for hCG we produced two additional substrates where the Ser in the P6 position was exchanged for a Val or an Asp (VV**SV**SEVL to VV**VV**SEVL, VV**DV**SEVL). A marked drop in activity was observed for both of these two new substrates with hCG (Fig 10). A Ser in position P6 of a substrate appears to be of major importance for the cleavage by hCG indicating that residues relatively far from the P1 cleavage site can have major impact on cleavage efficiency of this enzyme (Fig 10). The importance of a Ser in position P6 was not possible to extract from the phage display data as the variable region of the phage only is 9 amino acids long and cleavage often occur in the middle of this region, making predictions of specificity only possibly up to position P4.

### Analysing the effect of human lactoferrin on the cleavage activity of hCG

**Fig 11:**
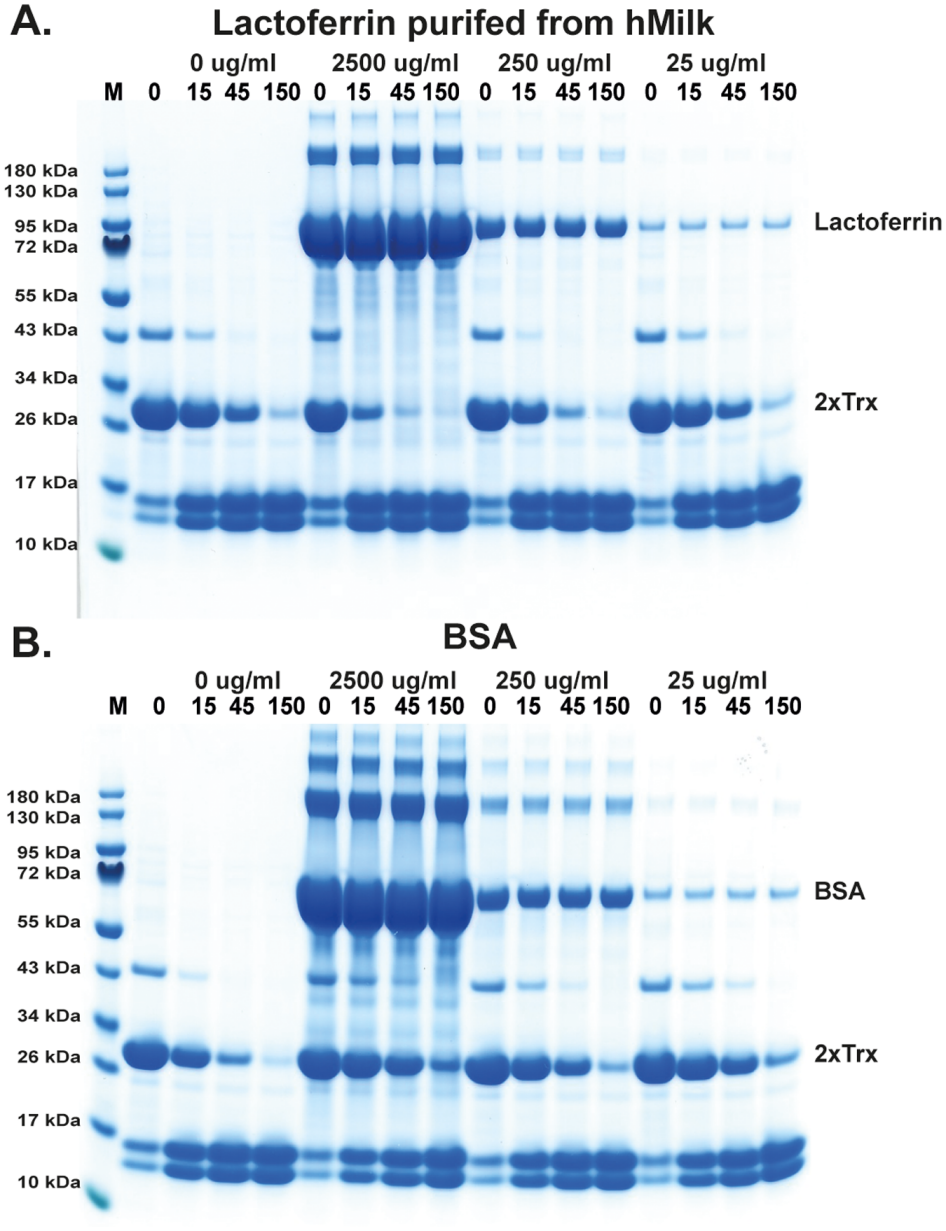
Analysis of the effect of human lactoferrin on the activity of hCG. The effect on the activity by hCG on the HC consensus substrate (VVLFSEVL) was analysed in a cleavage reaction as before (Figs 5–9). In panel A three different concentrations of lactoferrin were used: 25, 250 and 2500μg/ml. A 3–5 fold enhancement was observed at the highest lactoferrin concentration (2500μg/ml) and a 2–3 fold enhancement at the middle concentration (250μg/ml) but no enhancement at the low (250μg/ml) lactoferrin concentration. In panel B, bovine serum albumin (BSA) was used as a control to ensure that the enhancing effect was not simply an effect of higher protein concentration and thereby a stabilizing effect on hCG, but a specific lactoferrin dependent effect. As seen in panel B, no enhancement but instead a slight reduction in the cleavage of the recombinant substrate was observed by the addition of BSA.

In a recent study by Eipper et al they show that human lactoferrin can enhance the activity of hCG by a factor of 5–7 [47]. In their study they used an active site binding assay to assess the activity of hCG. In order to confirm and possibly get a better understanding of this enhancing effect we used the recombinant 2xTrx substrates to assay the potential activating effect by lactoferrin on hCG. By adding lactoferrin into the cleavage reaction at 25 μg/ml, 250 μg/ml or 2500 μg/ml, we could show in our assay system that there was a 3–5 fold enhancement in cleavage activity on hCG by lactoferrin but not of an unrelated protein such as bovine serum albumin (BSA) (Fig 11A and 11B). However, the enhancement was only observed at the two highest concentrations of lactoferrin, 250 and 2500 μg/ml (Fig 11A). Here the molar ratio between lactoferrin to hCG was approximately 37 and 370. The enhancing effect was also only 3–5 fold even at the highest lactoferrin concentration and no enhancement was seen at the low concentration of lactoferrin despite having an excess of lactoferrin to hCG of 3.6 times.

### Analysing the difference in efficiency and reliability of combinatorial peptide library with mass spectrometry to determine the extended cleavage specificity

In two recent studies by O’Donoghue et al., a novel method to study the extended specificities of some pure pathogen and immune related proteases and proteases in mixtures has been developed [35, 36]. This new method is based on a library of 14 amino acid long peptides where the products after cleavage by the analysed proteases are then subject to mass spectroscopic techniques (MS). This is a relatively rapid analysis, which may become an interesting alternative to phage display.

**Fig 12:**
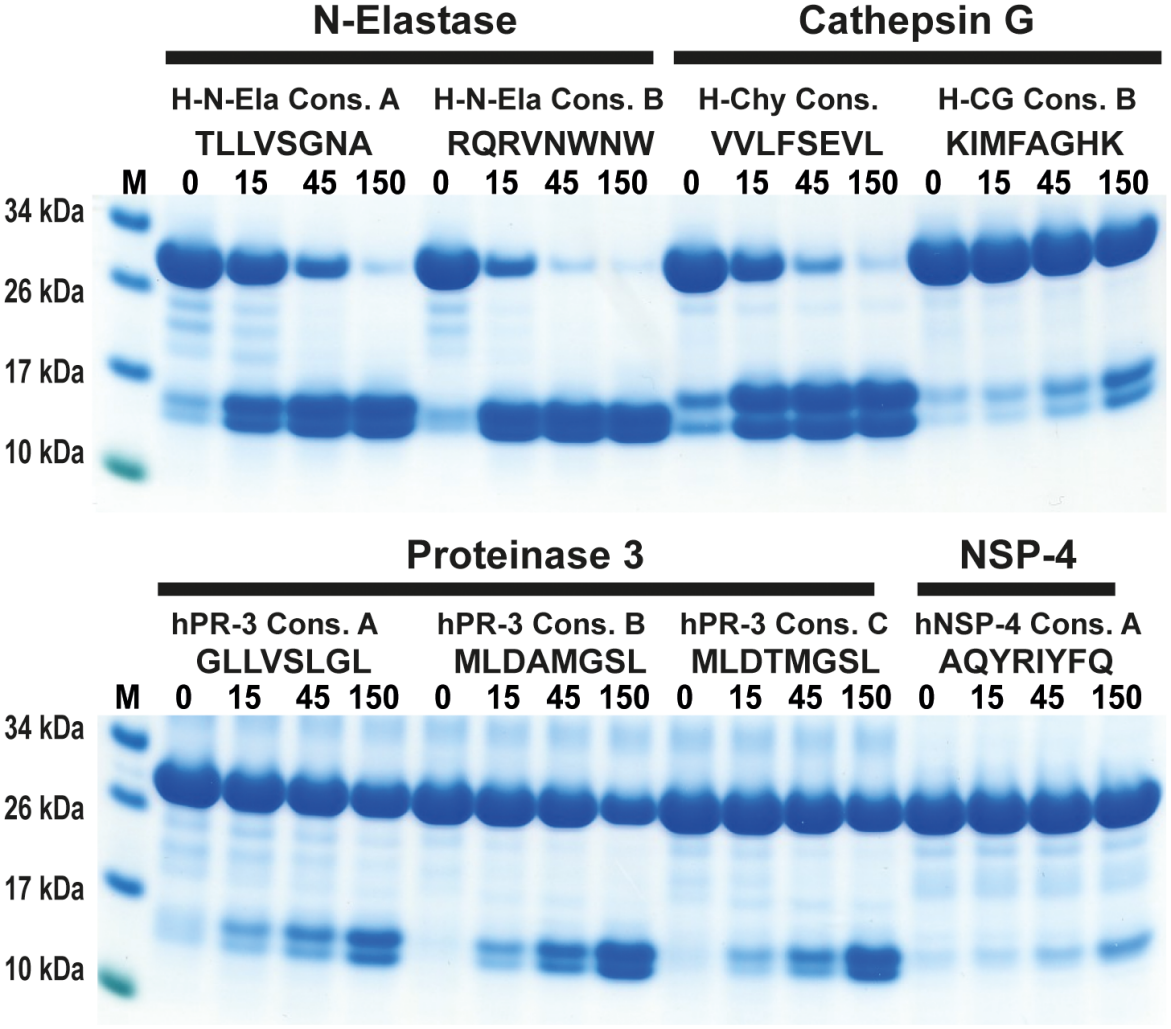
Analysis of the cleavage of a panel of consensus substrates for four human neutrophil proteases. The cleavage of a number of consensus cleavage sites for four different human neutrophil proteases (N-elastase, hCG, hPR3 and NSP4) were studied with the 2x-Trx system. The ‘A’ consensus sites come from phage display analyses performed in our lab and the substrates B and C comes from a proteomics study by O’Donoghue et al [36]. The substrates originating from the two different methods for N-elastase and proteinase 3 both show very good cleavage, whereas for hCG the consensus sites obtained by the proteomics method shows only minor cleavage, indicating a relatively poor site. No phage display has yet been performed on hNSP4, therefore only a proteomics site was studied where minor cleavage after using a relatively high concentration of the enzyme was seen.

As we now have a good consensus substrate for hCG and have also recently studied two other human neutrophil proteases (hNE and hPR3) by phage display (unpublished results), we can now compare the two methods. The consensus sequences from both methods were produced in the 2xTrx system and used to study the efficacy in cleavage by the enzymes. In the MS-study non-natural amino acids were used such as nor-leucine, which complicates the comparison. The most similar natural amino acids are methionine or leucine. We therefore inserted Met in the positions of the MS consensus sequences where they had used nor-leucines. As seen from Fig 12, the consensus sequences for both methods worked fine with the relatively unspecific enzymes hNE and hPR3. However for the more specific protease hCG the phage display sequence was clearly superior. Only minor cleavage was observed with the consensus sequence obtained by the proteomics method. This indicates that the proteomics method can be a method of choice for proteases with a broad specificity but not for more specific proteases where the number of cleavable peptides in the starting pool needs to be high. The substrate identified for NSP-4 by the MS technology was also a very poor substrate as we even after using a relatively large amount of enzyme only observed minor cleavage, which indicates that also NSP-4 is a relatively specific protease. In the proteomic studies only 124 originating peptides were used whereas the phage display library contains approximately 50 million different nine amino acid long sequences, indicating that for more specific proteases a larger starting complexity is required. With both technologies the cleavage can occur at multiple positions which results in 50 million + and 124 + potential cleave sites. However, one very positive characteristic of the proteomics method is the identification of substrates that are not optimal but still cleaved relatively efficiently, such as the cleavage of threonine containing substrates by hPR3 and lysine containing substrates by hCG, which had not been observed during the phage display screening as this method primarily identifies the most optimal substrates.

## Discussion

The granule associated serine proteases of the neutrophils have been studied quite extensively for many years, however, their extended cleavage specificities have never been studied in detail [9–12, 32]. The fine specificity of the enzymes and their selectivity for the different potential *in vivo* substrates they may encounter is to a large extent determined by their extended specificity, which is why such information can be of major importance for the biological functions of these enzymes. A detailed map of their extended specificity can also aid our search for their most important *in vivo* substrates.

Different methods can be used to study the extended specificity of an enzyme. The classical chromogenic substrates usually only cover the N terminal side of the cleavage sites whereas FRET peptides, peptide libraries and phage display can cover larger regions of the site including both N and C terminal sequences thereby providing a more complete view of the full extended specificity. Recently a novel proteomics method has also been developed to study protease specificity. This technique has been used to study the involvement of the different neutrophil proteases in the formation of NETS. Here, the authors concluded that N-elastase shows the most enzyme activity in the NETS [36]. Based on our analysis of the consensus cleavage sites identified by the proteomics and phage display studies we can now conclude that both techniques gave similar and accurate consensus sites for the relatively unspecific proteases such as N-elastase and proteinase 3. However, for the more specific proteases the complexity of the peptide library used for the proteomics study was most likely too limited to give a reliable result. The consensus sites for hCG by the proteomics method were suboptimal and only minor cleavage was seen with these substrates in the 2xTrx analysis (Fig 12). This result also indicates that the protease activity of the NETS may need to be re-evaluated. The peptide substrates for the latter enzyme in the proteomics study were not optimal, which may explain why its activity in the NETS was most likely underestimated. The analysis of the consensus sequences for the two techniques also indicates that the low complexity of the peptide library in the proteomics study with only 124 different peptides may limit its use for the analysis of more specific proteases. However, for relatively unspecific and complex mixtures of proteases it may be a very interesting alternative to phage display. Interestingly also the proteomics method identified Thr as a good P1 residue for hPR3, which has not been observed with phage display. If the proteomics study increased the number of peptides by a factor of 10 and did not use non-natural amino acids as for example nor-Leucine the technique may become a valuable addition to the methods used to determine the specificity of also more specific proteases in a near future. The use of natural amino acids only would also make the results more easy to confirm and validate with other non-MS based methods.

The analysis of the hCG presented in this communication now shows that this enzyme has an extended specificity that is very similar in many aspects to the HC. These two enzymes are encoded from the same chromosomal locus, the chymase locus, and likely originate from the same original gene via gene duplication [48–51]. Both lack the cysteine bridge Cys191/Cys220 (chymotrypsinogen numbering) that is present in most non hematopoetic serine proteases, which is thought to open up the catalytic site and possibly facilitate a broader specificity of these enzymes [32]. Our analysis of hCG now shows that hCG, like the HC, has a preference for negatively charged amino acids in the P2´position, a preference for aliphatic amino acids both N and C terminal of the cleavage site, and also efficiently cleaves sites with a Leu in the P1 position. The previously identified dual specificity, being both a chymase and a tryptase-type, has also been verified here and we can now show that the chymase activity is only 2 fold higher for the most optimal substrates, containing a Phe in the P1 position, compared to the Lys containing tryptase substrates. However, the activity on an Arg containing substrate was approximately 10 times lower than the chymase activity indicating a strong preference for Lys for tryptase activity, as previously indicated [31–33]. Interestingly, the activity on Trp containing substrates was highly dependent on positions surrounding the cleavage site. Very low activity was observed for the HC on the opossum chymase consensus substrate, which contains a Trp in the P1 position, whereas the substrate with multiple Val residues in P2, P3 and P4 positions showed no difference between Phe, Tyr and Trp containing substrates (Figs 5 and 6). Here a clear difference was observed between the two enzymes. With the VVV substrates, hCG showed a preference for Phe over Tyr by a factor 2 and over and Trp by a factor 3–5. In contrast, the activity on Leu containing substrates was very similar for the two enzymes, approximately only 2 times lower than the optimal Phe substrate (Fig 6C and 6D). It was noteworthy that these proteases showed a marked difference between their overall activity. The difference was approximately 10-fold on a molar basis. This lower activity of hCG has previously also been observed and proposed to be dependent on the widening of the active site, which results in a broader specificity, being both chymase and tryptase-type, but at the same time lowering the affinity to the substrates and thereby lowering cleavage rate [32].

We have previously shown that the preference for negatively charged amino acids in the P2´position for the HC is governed by Arg143 and Lys192 [40]. They both contribute to this preference to approximately the same extent [40]. We now observe that hCG also shows a similar preference for negatively charged amino acids in the P2´position (Figs 3 and 5). Analysis of its primary amino acid sequence shows that hCG also has positively charged amino acids in the same positions of the enzyme indicating that the Arg143 and Lys192 could be responsible for this preference in target sequences (data not shown). Interestingly, the preference was more obvious for both enzymes when using substrates with a Gly in the P2´position compared to Ala where only a minor difference to Asp could be observed (Fig 7A and 7B). A preference for acidic residues in P2´ position has also been observed for the opossum α-chymase, rMCP-5 and mMCP-4 [34, 46, 52]. All of these chymases contain Arg143 and Lys192 residues. Other chymases that also have Arg143 and Lys192 are the α-chymases from the crab eating macaque and baboon, the sheep mast cell chymase, mMCP-5, and hamster chymase-2. Furthermore, the β-chymases rMCP-3, hamster chymase-1, the guinea pig α-chymase and gerbil chymase-1 also contain Arg143 and Lys192 [53]. The preference for acidic P2´ residues seems to be highly preserved among the α-chymases. However, there are exceptions. The dog α-chymase holds Lys143 and Lys192 residues, and rMCP-1 holds Gln143 and Lys192 and both of these enzymes have no or only very weak preference for acidic residues in P2´ [34, 39]. Apparently, a minor change from the positively charged Arg to a slightly smaller but still positively charged side chain of a Lys residue in position 143 is enough to partially affect the preference for acidic P2´ residues. The gerbil chymase 2 also holds Lys143 and Lys 192 and may therefore also have a lower preference for acidic aa in the P2’ position [54].

The strong activity of HC on P1 Leu containing recombinant substrates in Figs 6C and 9B was unexpected based on the results from the chromogenic substrate assay and the phage display (Figs 2 and 3). This finding indicated a marked difference in the activity on short truncated substrates as represented by the chromogenic substrates, which lack amino acids C-terminal of the cleavage site. The smaller amino acid Leu compared to the aromatic Phe and Tyr may therefore need additional support by residues both upstream and downstream of the cleavage site for proper positioning and binding to the active site of the enzyme. The very large difference on the cleavage rate of a substrate by relatively similar amino acids surrounding the cleavage site was also somewhat unexpected. This was typified in the activity of two Trp containing substrates where the opossum consensus (VGLWLDRV) was a poor substrate for HC (Fig 5C) whereas the VVVWSEVV substrate was as good as the Phe consensus substrate for this enzyme (Fig 6C). In contrast hCG was less active on both of these Trp containing substrates (Figs 5 and 6). The notable effect by Ser in position P6 was also unexpected and differed between the two enzymes where only hCG showed this marked preference (Fig 10). This latter finding closely resembles the situation seen for thrombin, another member of the chymotrypsin gene family [43]. Thrombin is for some substrates highly dependent in its cleavage activity of sites located either close or further away from the active site. The fibrinogen alpha chain use a region located just upstream of the cleavage site to enhance cleavage of a site that is relatively poor in the absence of this region. This enhancing site is located only 4–8 amino acids upstream of the P1 residue [43]. In contrast, cleavage sites for thrombin within coagulation factors V and VIII such enhancing regions extend more than 100 amino acids upstream of the cleavage site and these regions interact with positively charged regions on the enzyme, so called exosites [43]. Here hCG showed a tendency of this more local interaction when the cleavage was dependent on a site located 6 amino acids upstream of the actual cleavage site.

Recently the activity of hCG has been shown to be modulated by other granule stored proteins. Lactoferrin is an iron binding protein stored in the neutrophil granules, which can enhance activation of hCG [47]. The activity of the enzyme can be increased by a factor of 5–7 with the addition of lactoferrin [47]. Similarly to the cooperation seen with lactoferrin and hCG, human thrombin has interactions with other proteins when cleaving protein C. Thrombin is a potent activator of protein C by cleaving an activation site, which is highly dependent on the accessory molecule thombomodulin. In order for this to efficiently occur, thrombomodulin needs to be bound to the complex. Despite this, the exact mechanism of protein C activation is not known and in this regard the situation for hCG and lactoferrin is also, to our knowledge, not fully elucidated. However, both of these examples highlight the very complex nature sometimes involved in substrate interactions and cleavage. Here we could confirm the enhancing activity of lactoferrin on hCG by incubating lactoferrin and recombinant 2x Trx substrates with hCG (Fig 11A). However, the effect was only seen with an excess of lactoferrin to hCG with the highest concentrations corresponding to 37 and 370 times more lactoferrin to hCG, indicating that the effect is not necessarily direct specific binding, which is generally an equimolar interaction, but more dependent on other unknown factors.

Other factors such as the accessibility of the potential cleavage site can become more relevant than the sequence itself. A relatively poor site can be cleaved relatively efficiently when fully exposed, whereas an ideal consensus site, when being well hidden in the structure, might not be cleaved at all even in the presence of the enzyme at a high concentration. In that context, initial cleavages might also facilitate access to otherwise non-exposed sites. Taken together, in order to fully address protease’s accessibility to a potential cleavage site or sites, any proposed substrate needs to be tested experimentally. This is highlighted in a recent study where the cleavage sensitivity of 50 different human cytokines and chemokines for cleavage by the HC and hCG was tested. The general view suggested that the HC and hCG would cleave a broad range of targets yet, remarkable, in this study only a few cytokines were cleaved despite most of them contained what appeared to be close to optimal sites for HC or hCG [55]. Only 4 out of 50 cytokines tested were cleaved by the HC, with a few additional by hCG but only under equimolar substrate to enzyme ratios [55]. This highlights factors such as accessibility of the site as being of major importance for actual cleavage.

Among the targets identified in above study included several alarmins, such as IL-18 and IL-33, suggesting a role for these two enzymes in limiting excessive inflammation [55]. A number of additional potential targets have also been identified for both of these enzymes. Angiotensin (Ang) is a well studied target where Ang I is cleaved efficiently by both HC and hCG resulting in a product Ang II, an active peptide involved in blood pressure regulation [56–58]. Interestingly hCG is relatively resistant to inactivation by plasma protease inhibitors (serpins) when bound to the neutrophil cell surface [59]. This is in contrast to when it is free in plasma and can possibly explain why surface bound hCG can act as a potent Ang II converter even in the presence of inhibitors [59]. For many of the potential targets the difference between HC and hCG activities is most likely due to the broader specificity of hCG compared to the HC. The relatively strong tryptase activity of hCG most likely results in the capacity of this enzyme to cleave a broader range of substrates. However, the lower overall enzymatic activity of hCG also needs to be taken into account when analysing its in vivo function. Here there is the possibly enhancing activity in the presence of lactoferrin, which could have an important role in its regulation. Despite this the potentiating activity was only 3–5 fold higher in our assay even at a very high molar excess of lactoferrin, indicating only a modest enhancement under physiological conditions.

In summary, we demonstrate here that hCG shows some enzyme characteristics that are closely comparable to HC. Both enzymes are chymase-type proteases with a preference for Phe and Tyr over Trp in the P1 position of substrates as well as they show potent activity toward Leu containing substrates. However, they differ in aspects relating to their tryptase activity where hCG readily cleaves Lys containing substrates in the P1 position whereas HC only shows a very weak activity against Arg and no activity towards Lys. Somewhat surprisingly, the phage display analysis of hCG did not select any tryptase-like targets, which suggests that the technique can primarily be used to identify the most optimal targets. Targets that are cleaved only 2–3 times less efficiently than the optimal ones are apparently primarily selected against during the panning. This needs to be taken into consideration when analysing the specificity of enzymes so that significant secondary specificities would not be overlooked. hCG also has an overall much lower enzymatic activity, approximately 10-fold, as compared to HC. These two enzymes also differ in the preference for the amino acids in the P1 position where negatively charged residues are strongly non-preferred by hCG but are favoured for HC. Interestingly there is also a strong preference for Ser in the P6 position for hCG but not for HC. This is an indication that for positions relatively far from the active site can be of major importance for the cleavage, not only for thrombin exosite interactions but also for the granule stored serine proteases of hematopoietic cells.

We hope that the extended specificities presented here for both hCG and HC can now be used as a refined tool to identify and validate old and potentially new *in vivo* targets for these two important immune proteases. As we increase our understanding of the function of these proteases and identify new targets, both of endogenous and exogenous (viral and bacterial) origin, this knowledge hopefully will facilitate the identification of important immunomodulatory or antimicrobial targets for treatment.

## Experimental procedures

### Production and purification of proteases

The hCG used in this study had been purified from peripheral blood neutrophils and was purchases from BioCentrum (Krakow, Poland). Human proteinase 3 and N-elastase were also purified from peripheral blood neutrophils and purchased from Lee Biosolutions (St. Louis, Missouri, USA) and Athens Research & Technology (Athens, GA, USA), respectively. The constructs and procedure for the production of recombinant HC from bacculovirus infected insect cells has previously been described in detail [37]. Human activated plasma thrombin, purchased from Sigma Aldrich (Sigma T-6884), and human granzyme B produced in-house in the human cell line HEK-293 EBNA using the vector pCEP-Pu2 [40] were used in the chromogenic substrate assay.

Protein purity and concentration was estimated by separation on 12.5% SDS-PAGE gels. Protein samples were mixed with sample buffer, and β-mercapto-ethanol was added to a final concentration of 5%. To visualize the protein bands, the gel was stained with colloidal Coomassie Brilliant Blue [60].

### Analysis of primary specificity by cleavage of chromogenic substrates

Enzymatic activity was measured towards a panel of chromogenic substrates. These substrates were purchased from Bachem (Bubendorf, Switzerland) and Chromogenix (Mölndal, Sweden). Measurements were performed in 96-well microtiter plates with a substrate concentration of 0.18 mM in 200 μl PBS. Hydrolysis was monitored spectrophotometrically at 405 nm for up to 10 hrs in a Versamax microplate reader (Molecular Devices, Sunnyvale, CA).

### Determination of cleavage specificity by phage-displayed nonapeptide library

A library of 5x10^7^ unique phage-displayed nonameric peptides was used to determine the cleavage specificity of the hCG as previously described [34, 52, 61]. In these T7 phages, the C-terminus of the capsid protein 10 were manipulated to contain a nine aa long random peptide followed by a His_6_-tag [61]. An aliquot of the amplified phages (~10^9^ pfu) were bound to 100 μl Ni-NTA beads by their His_6_-tags for 1 h at 4°C under gentle agitation. Unbound phages were removed by washing ten times in 1.5 ml 1 M NaCl, 0.1% Tween-20 in PBS, pH 7.2, and two subsequent washes with 1.5 ml PBS. The beads were finally resuspended in 1 ml PBS. Activated and heparin-Sepharose purified HC mutant (~0.1 μg) was added to the resuspended beads and left to digest susceptible phage nonapeptides under gentle agitation at room temperature over night. PBS without protease was used as control. Phages with a random peptide that was susceptible to protease cleavage were released from the Ni-NTA matrix, and the supernatant containing these phages was recovered. To ensure that all of the released phages were recovered the beads were resuspended in 100 μl PBS (pH 7.2) and the supernatant, after mixing and centrifugation, was added to the first supernatant. To ensure that the His_6_-tags had been hydrolyzed on all phages recovered after protease digestion, 15 μl fresh Ni-NTA agarose beads were added to the combined phage supernatant and the mixture agitated for 15 min followed by centrifugation. A control elution of the phages still bound to the beads, using 100 μl 100 mM imidazole showed that at least 1 x 10^8^ phages were attached to the matrix during each selection. Ten μl of the supernatant containing the released phages was used to determine the amount of phages detached in each round of selection. Dilutions of the supernatant were plated in 2.5 ml of 0.6% top agarose containing 300 μl of *E*. *coli* (BLT5615), 100 μl diluted supernatant and 100 μl 100mM IPTG. The remaining volume of the supernatant was added to a 10 ml culture of BLT5615 (OD ~0.6). The bacteria had 30 min prior to phage addition been induced to produce the T7 phage capsid protein by the addition of 100 μl 100 mM IPTG to the culture. The bacteria lysed approximately 75 minutes after phage addition. The lysate was centrifuged to remove cell debris and 500 μl of the phage sub-library was added to 100 μl fresh Ni-NTA beads, to start the next round of selection. After binding the sub-library for 1 h at 4°C under gentle agitation, the Ni-NTA beads were washed 15 times in 1.5 ml 1 M NaCl, 0.1% Tween-20 in PBS, pH 7.2, followed by two subsequent washes with 1.5 ml PBS.

Following five rounds of selection, 100 plaques were isolated from LB plates after plating in top agarose. Each phage plaque, corresponding to a phage clone, was dissolved in phage extraction buffer (100 mM NaCl and 6 mM MgSO_4_ in 20 mM Tris-HCl pH 8.0) and vigorously shaken for 30 min in order to extract the phages from the agarose. The phage DNA was then amplified by PCR, using primers flanking the variable region of the gene encoding the modified T7 phage capsid-protein. After amplification, 96 PCR fragments were sent unpurified to GATC Biotech Germany for sequencing.

### Generation of a consensus sequence from sequenced phage inserts

Phage insert sequences were aligned by hand assuming a preference for aromatic aa in position P1. Sequences with only one aromatic aa were aligned first and sequences with more than one possible cleavage site were then aligned to fit this pattern. Amino acids with similar characteristics were grouped together as follows: aromatic (Phe, Tyr, Trp); negatively charged (Asp, Glu); positively charged (Lys, Arg); small aliphatic (Gly, Ala); larger aliphatic (Val, Leu, Ile, Pro); hydrophilic (Ser, Thr, His, Asn, Gln, Cys, Met). The nomenclature by Schechter and Berger [62] was adopted to designate the aa in the substrate cleavage region, where P1-P1’ corresponds to the scissile bond.

### Generation of recombinant substrates for the analysis of the cleavage specificity

A new type of substrate was developed to verify the results obtained from the phage display analysis. Two copies of the *E*. *coli* thioredoxin gene were inserted in tandem into the pET21 vector for bacterial expression (Fig 5A). In the C-terminal end a His_6_- tag was inserted for purification on Ni^2+^ IMAC columns. In the linker region, between the two thioredoxin molecules, the different substrate sequences were inserted by ligating double stranded oligonucleotides into two unique restriction sites, one BamHI and one SalI site (Fig 5A). The sequences of the individual clones were verified after cloning by sequencing of both DNA strains. The plasmids were then transformed into the *E*.*coli* Rosetta gami strain for protein expression (Novagen, Merck, Darmstadt, Germany). A 10 ml overnight culture of the bacteria harbouring the plasmid was diluted 10 times in LB + Amp and grown at 37 °C for 1–2 hours until the OD (600 nm) reached 0.5. IPTG was then added to a final concentration of 1 mM. The culture was then grown at 37°C for an additional 3 h under vigorous shaking, after which the bacteria were pelleted by centrifugation at 3500 rpm for 12 minutes. The pellet was washed once with 25 ml PBS + 0.05% Tween 20. The pellet was then dissolved in 2 ml PBS and sonicated 6 x 30 seconds to open the cells. The lysate was centrifuged at 13000 rpm for 10 minutes and the supernatant was transferred to a new tube. Five hundred μl of Ni-NTA slurry (50:50) (Qiagen, Hilden, Germany) was added and the sample was slowly rotated for 45 min at RT. The sample was then transferred to a 2 ml column and the supernatant was allowed to slowly pass through the filter leaving the Ni-NTA beads with the bound protein in the column. The column was then washed four times with 1 ml of washing buffer (PBS + 0.05% Tween + 10 mM Imidazole + 1 M NaCl). Elution of the protein was performed by adding 150 μl elution buffer followed by five 300 μl fractions of elution buffer (PBS + 0.05% Tween 20 + 100 mM Imidazole). Each fraction was collected individually. Ten μl from each of the eluted fractions was then mixed with 1 volume of 2 x sample buffer and 1 μl β-mercapto-ethanol and then heated for 3 min at 80°C. The samples were analysed on a SDS bis tris 4–12% PAGE gel and the second and third fractions that contained the most protein were pooled. The protein concentration of the combined fractions was determined by Bio-Rad DC Protein assay (Bio-Rad Laboratories Hercules, CA USA). Approximately 60 μg of recombinant protein was added to each 120 μl cleavage reaction (in PBS). Twenty μl from this tube was removed before adding the enzyme, the 0 minute time point. The active enzyme was then added (approximately 35 ng of the HC or 350 ng of hCG) and the reaction was kept at room temperature during the entire experiment. Samples (20 μl) were removed at the indicated time points (15 min, 45 min and 150 min) and stopped by addition of one volume of 2 x sample buffer. β-mercaptoethanol (1 μl) was then added to each sample followed by heating for 3 min at 80 °C. Samples (20 μl each) were then analysed on 4–12% pre-cast SDS-PAGE gels (Invitrogen, Carlsbad, CA, USA). The gels were stained overnight in colloidal Coomassie staining solution and de-stained for several hours according to previously described procedures [60]. Some of the most important protein gels were analyzed using the UN-SCAN-IT Gel Analysis Software from Silk Scientific Inc. (Orem, Utah USA). The gel pictures had first to be turned into grey scale pictures before the densitometric analysis. The scanning results are presented as separate panels in Figs 5, 6 and 8.

## Abbreviations

aa: amino acid(s)
Ang: angiotensin
HC: human chymase
hCG: human cathepsin G
Ni-NTA: nickel-nitrilotriacetic acid
